# The Population Genetics, Virulence, and Public Health Concerns of *Escherichia coli* Collected From Rats Within an Urban Environment

**DOI:** 10.3389/fmicb.2021.631761

**Published:** 2021-10-28

**Authors:** J. B. Pettengill, J. A. Kase, M. H. Murray

**Affiliations:** ^1^Division of Biostatistics and Bioinformatics, Office of Analytics and Outreach, Center for Food Safety and Applied Nutrition, United States Food and Drug Administration, College Park, MD, United States; ^2^Division of Microbiology, Office of Regulatory Science, Center for Food Safety and Applied Nutrition, United States Food and Drug Administration, College Park, MD, United States; ^3^Davee Center for Epidemiology and Endocrinology, Urban Wildlife Institute, Lincoln Park Zoo, Chicago, IL, United States

**Keywords:** *Escherichia coli*, rats, virulence, food safety, urban ecology, population genetics

## Abstract

The co-existence of rats and humans in urban environments has long been a cause for concern regarding human health because of the potential for rats to harbor and transmit disease-causing pathogens. Here, we analyze whole-genome sequence (WGS) data from 41 *Escherichia coli* isolates collected from rat feces from 12 locations within the city of Chicago, IL, United States to determine the potential for rats to serve as a reservoir for pathogenic *E. coli* and describe its population structure. We identified 25 different serotypes, none of which were isolated from strains containing significant virulence markers indicating the presence of Shiga toxin-producing and other disease-causing *E*. *coli*. Nor did the *E. coli* isolates harbor any particularly rare stress tolerant or antimicrobial resistance genes. We then compared the isolates against a public database of approximately 100,000 *E. coli* and *Shigella* isolates of primarily food, food facility, or clinical origin. We found that only one isolate was genetically similar to genome sequences in the database. Phylogenetic analyses showed that isolates cluster by serotype, and there was little geographic structure (e.g., isolation by distance) among isolates. However, a greater signal of isolation by distance was observed when we compared genetic and geographic distances among isolates of the same serotype. This suggests that *E. coli* serotypes are independent lineages and recombination between serotypes is rare.

## Introduction

The co-existence of rats and humans within densely populated urban areas has long been an issue of concern for human public health. A primary reason for concern is rats (e.g., *Rattus norvegicus* and *Rattus rattus*) serving as a vector and reservoir for human disease-causing pathogens including zoonotic bacteria (e.g., *Yersina pestis* and *Leptospira interrogans*), viruses (e.g., Seoul hantavirus), and parasites (Himsworth et al., 2013). Rats may also transmit antimicrobial resistant strains of various pathogens (e.g., Extended-spectrum beta-lactamase producing *E. coli*, Guenther et al., 2012) and cause substantial economic losses (Stenseth et al., 2003; Byers et al., 2019). As a result, municipalities allocate substantial resources to control rat populations with the assumption that removing the rats will remove disease-causing pathogens.

Here, we are interested in the potential for rats to cause public health issues within the food supply through the transmission of harmful *E. coli*, which is often associated with food-poisoning (Centers for Disease Control Prevention, 2020). A key factor in determining the public health risk posed by rats is whether rats actually harbor pathogenic *E. coli*, as opposed to commensal *E. coli*. Evidence suggests that rats are likely to carry *E. coli*, but potentially not O157 Shiga Toxin *E. coli* (STEC) or other pathogenic strains [e.g., enteropathogenic *E*. *coli* (EPEC), enteroinvasive *E*. *coli* (EIEC), and uropathogenic *E. coli* (UPEC)]. For example, 84% of rats sampled in a study in Trinidad and Tobago carried *E. coli* but none were of serogroup O157 or hemolytic on blood agar (Nkogwe et al., 2011). In a study of urban rats within the city of Berlin, Germany, *E. coli* strains from *R. norvegicus* did not harbor the genes often associated with highly virulent STEC [e.g., Shiga toxin genes 1 (*stx1*) or 2 (*stx2*) or the adherence factor intimin (*eae*) gene] (Guenther et al., 2012).

Another important factor in assessing the public health risks posed by even commensal *E. coli* within urban environments is the extent to which they carry antimicrobial resistant genes (AMR). Furness et al. (2017) found that small mammals may be useful indicators of the variation in AMR within the environment. Other studies of small mammals including rats have found that the incidence of antibiotic resistant *E. coli* in such animals is higher in areas close to livestock compared to wild environments suggesting a mechanism for transmission of AMR (Kozak et al., 2009; Nhung et al., 2015). However, studies investigating the incident of antibiotic resistant *E. coli* from small mammals within urban environments is lacking.

Also of importance in determining the public health concern is the population genetic structure of *E. coli* in urban rats as it can help inform eradication efforts. For example, if rats harbor pathogenic *E. coli* and the microbial population genetic diversity is highly structured and heterogenous then epidemiological and control measures may too need to be heterogenous to account for local conditions (Angley et al., 2018). A model described as best suited to *E. coli* population genetic diversity is one in which there are distinct non-recombining lineages (e.g., serotypes), within which recombination is more likely to occur (Ochman and Selander, 1984). With the advent of whole-genome sequencing (WGS) a better understanding has been gained regarding the contradiction between clonality and recombination within *E. coli* (Tenaillon et al., 2010). Further refinements of the historical model propose that *E. coli* population genetic structure is defined best by predominant clonal evolution (PCE) and “near-clading” (i.e., clonal lineages within which there are discrete subdivisions) (Tibayrenc and Ayala, 2017). This leads to the possibility that isolation by distance at the species level (a positive correlation between genetic and geographic distances) is unlikely to be observed but such a pattern may exist within serotypes.

Here we present an investigation of *E. coli* that were found within rats located throughout the metropolitan area of Chicago, IL, United States. We used whole-genome sequence data to predict serotypes, virulence, and antimicrobial resistance and to describe the public health risks posed by this bacterium within rats. We also performed phylogenetic and population genetic analyses to understand the diversity and structure of *E. coli* within the rats. We predicted that *E. coli* population structure would be consistent with isolation-by-distance because this pattern has been detected in rat population structure in multiple cities (Combs et al., 2018). Lastly, we discuss these results in the light of current thinking on the genetic structure of this pathogen, the likelihood that it warrants concern as a vector of contamination of harmful *E. coli* into the food supply, and whether rat eradication efforts are likely to simultaneously control commensal organisms.

## Materials and Methods

### Study Area, Sample Collection, and Whole-Genome Sequencing

**FIGURE 1:**
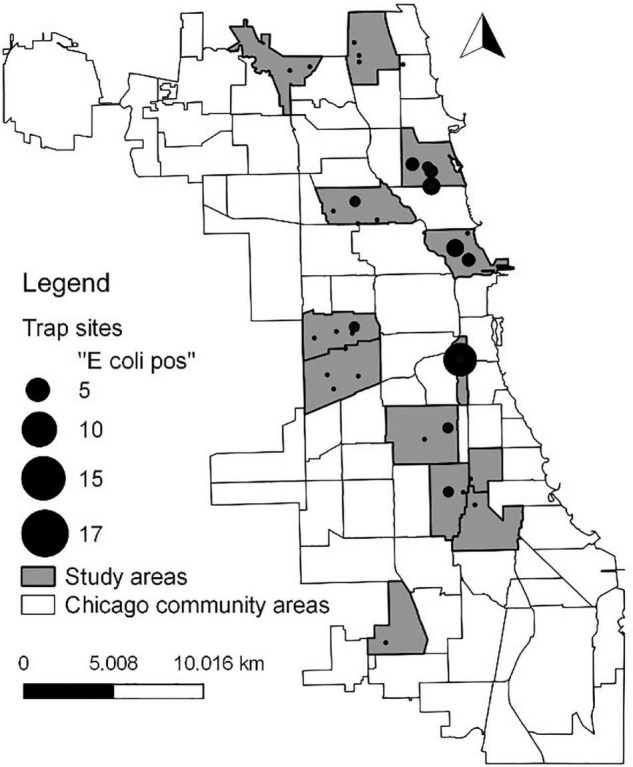
Map showing locations of *E. coli* isolates. Circle size is proportional to the number of isolates.

We collected *E. coli* from rat populations distributed throughout 12 different communities in the city of Chicago, Illinois (Figure 1), which is the third largest city in the United States (estimated population size of 2.7 million). As in most cities, the size of the rat population in Chicago is unknown but the number of rat complaints made by Chicago residents has increased in recent years (Murray et al., 2018). To sample rats throughout the city, the 12 communities were chosen to represent gradients in household income and 311 rat complaints. Within each community, the four alleys with the highest number of rat complaints were selected for trapping of rats. See Murray et al. (2018, 2020) for additional details on the communities targeted.

We partnered with Landmark Pest Management company to collect trapped rats within the targeted communities. Traps were set following a standardized protocol with 10 traps per alley for a one-month period throughout 2018 to minimize longer-term variation in rat population structure. Trapped rats were immediately frozen at −20°C until processing. Rats were thawed to 4°C and necropsied following standard procedures (Fiette and Slaoui, 2011). We collected colon contents for aerobic bacterial culture of *E. coli*, *Salmonella enterica*, *Campylobacter* sp., and *Staphylococcus aureus*. Biological samples were stored at −80°C in sterile Whirlpak bags until they were shipped to the Wyoming State Veterinary Laboratory for microbiological processing. Intestinal contents were plated for isolation with a sterile swab onto Columbia blood agar with 5% sheep blood (CBA), MacConkey (MAC), and Campy blood free Karmali (CAMPY) (Hardy Diagnostic, Santa Maria, CA). Subsequently, the swab was placed in selenite cystine broth (Hardy Diagnostics) for enrichment of *Salmonella* spp. The CBA, MAC plates, and selenite broth were incubated at 37°C in ambient O2. CAMPY plates were incubated at 41°C in microaerophilic conditions. At ∼18–24 h, the selenite broth was sub-cultured for isolation onto HardyCHROM Salmonella, and Hektoen Enteric agar plates (Hardy Diagnostic) and incubated at 37°C ambient O2. Culture plates were read and documented once at 18–24 h, and again at 36–48 h. A single colony from each sample were analyzed by matrix-assisted laser desorption/ionization time-of-flight mass spectrometry (Bruker Biotyper, Hamburg Germany) following the manufacturer’s instructions for identification. Isolates for identified taxa were sub-cultured and then frozen at -80°C for further analysis. We acknowledge that the sampling of a single colony does not permit investigation into the diversity of *E. coli* found within a single host.

Whole-genome sequencing was performed on all *E. coli* isolates using the Illumina MiSeq platform with the Nextera XT library prep kit and V2 chemistry. Sequencing was done according to the manufacturer’s protocols. All sequence data is publicly available in NCBI’s SRA archive; SRA accessions can be found in Table 1. Draft genome assemblies were created from the WGS data using the default settings in SKESA v.2.2 (Souvorov et al., 2018). All assemblies were evaluated to ensure they were of high quality (e.g., expected genome length, acceptable number of contigs, N50, etc.); multiple quality checks are also performed upon submission of the data to the public NCBI Pathogen Database. The program ectyper v0.8.1^1^ was used to predict the serotype from the draft assemblies for each isolate; to provide a measure of genetic differentiation within serotype we used mlst v2.16.1 (Seemann, 2020), which uses PubMLST (Jolley et al., 2018), to predict sequence type. Draft assemblies were annotated using PROKKA 1.12 (Seemann, 2014) with default settings.

**TABLE 1:** Sample information for the 41 *E. coli* isolates.

**Strain name**	**SRA accession**	**Predicted serotype**	**ST**	**Isolation date**	**Location**	**Latitude**	**Longitude**
CFSAN092693	SRR10094656	O10:H56	UNK	2018/11/30	Armour Square	41.8461725	−87.6344193
CFSAN085881	SRR8943499	O109:H21	155	2018/04/10	Englewood	41.780108	−87.620412
CFSAN085886	SRR8943510	O109:H21	155	2018/03/20	Logan Square	41.924763	−87.702318
CFSAN092692	SRR10095176	O129:H48	10	2018/11/30	Armour Square	41.8461725	−87.6344193
CFSAN085901	SRR8943412	O132:H49	1081	2018/05/15	Near North Side	41.8959207	−87.6287514
CFSAN085885	SRR8943503	O132:H49	1081	2018/03/12	Logan Square	41.914005	−87.700618
CFSAN085896	SRR9153371	O132:H49	1081	2018/05/23	North Lawndale	41.8602563	−87.7171906
CFSAN085906	SRR9155836	O132:H49	1081	2018/05/10	South Lawndale	41.8517183	−87.7110829
CFSAN085878	SRR8943521	O132:H8	155	2018/03/29	Edgewater	41.990916	−87.660294
CFSAN092694	SRR10095067	O150:H8	906	2018/11/19	Greater Grand Crossing	41.773795	−87.624554
CFSAN092690	SRR10094041	O153:H14	533	2018/11/21	Armour Square	41.8461725	−87.6344193
CFSAN085888	SRR8943492	O153:H14	533	2018/03/05	Lake View	41.932679	−87.651438
CFSAN085905	SRR9153345	O153:H14	533	2018/04/23	South Lawndale	41.831541	−87.716899
CFSAN085892	SRR8943111	O153:H30	38	2018/04/11	Lake View	41.939949	−87.651509
CFSAN085897	SRR8943397	O163:H14	5702	2018/03/23	Near North Side	41.901675	−87.635139
CFSAN092691	SRR10094015	O17:H41	7063	2018/11/26	Armour Square	41.8461725	−87.6344193
CFSAN092695	SRR10094646	O170:H28	UNK	2018/11/15	Lake View	41.9422062	−87.6539801
CFSAN085902	SRR8943512	O174:H2	661	2018/04/03	South Lawndale	41.831541	−87.716899
CFSAN085894	SRR8943105	O18:H49	6683	2018/04/17	North Lawndale	41.857211	−87.707848
CFSAN085903	SRR8943395	O2:H6	141	2018/04/17	South Lawndale	41.838146	−87.700414
CFSAN085875	SRR10094641	O32:H12	607	2018/05/07	Armour Square	41.8425421	−87.6376093
CFSAN085898	SRR8943396	O39:H7	6096	2018/03/23	Near North Side	41.901675	−87.635139
CFSAN085899	SRR8943405	O39:H7	6096	2018/03/23	Near North Side	41.901675	−87.635139
CFSAN085879	SRR8943404	O4:H5	UNK	2018/04/02	Edgewater	41.994818	−87.658589
CFSAN085891	SRR8943482	O4:H5	12	2018/04/21	Lake View	41.943418	−87.663917
CFSAN085877	SRR8943522	O45:H8	75	2018/05/07	Beverly	41.7049373	−87.6841668
CFSAN085893	SRR8943109	O62:H30	34	2018/04/11	Lake View	41.939949	−87.651509
CFSAN085876	SRR8943106	O7:H7	80	2018/05/07	Armour Square	41.8461725	−87.6344193
CFSAN085890	SRR8943197	O7:H7	80	2018/03/12	Lake View	41.932679	−87.651438
CFSAN085887	SRR8943501	O7:H7	80	2018/03/05	Lake View	41.932679	−87.651438
CFSAN085889	SRR8943485	O71:H12	5295	2018/03/05	Lake View	41.943418	−87.663917
CFSAN092688	SRR10094048	O71:H45	UNK	2018/11/14	Armour Square	41.8461725	−87.6344193
CFSAN085900	SRR8943406	O71:H45	UNK	2018/05/17	Near North Side	41.8959207	−87.6287514
CFSAN085874	SRR9153325	O8:H25	58	2018/05/23	Armour Square	41.8461725	−87.6344193
CFSAN085880	SRR8943401	O8:H8	5937	2018/04/10	Englewood	41.779811	−87.640871
CFSAN085882	SRR8943493	O8:H8	5937	2018/04/23	Forest Glen	41.9902171	−87.748391
CFSAN085904	SRR9153344	O8:H9	423	2018/04/17	South Lawndale	41.837282	−87.702103
CFSAN085884	SRR8943115	UNK	UNK	2018/03/12	Logan Square	41.924763	−87.702318
CFSAN085883	SRR8943117	UNK	UNK	2018/05/15	Forest Glen	41.9920822	−87.7350141
CFSAN085895	SRR8943523	UNK	UNK	2018/05/13	North Lawndale	41.862547	−87.703047
CFSAN085907	SRR9155721	UNK	UNK	2018/05/04	Washington Park	41.7867588	−87.6274017

### Phylogenetic, Population Genetic, and Recombination Analyses

An internal core-genome multi-locus sequence typing (cgMLST) scheme (Pightling et al., 2019) was used to identify homologous genes across the 41 annotated genomes. Each single-copy gene from each isolate was aligned using the program muscle v3.8.31 (Edgar, 2004) with the default settings. The cgMLST loci were concatenated into a single alignment from which variable positions (single nucleotide polymorphisms; SNPs) were extracted using a custom python script. A phylogenetic tree was inferred from the resulting SNP matrix using the program fasttree v2.1.11 (Price et al., 2010) with datatype and nucleotide substitution model set to nt and gtr, respectively.

To determine whether there was evidence for isolation by distance, as expected if *E. coli* in rats had a similar population expected of their hosts, we estimated pairwise genetic distances based on the SNP matrix from the concatenated cgMLST loci using the dist.dna function within the R package ape (Paradis et al., 2004) with model set to “N.” A corresponding pairwise geographic distance matrix was estimated using the distm function in the R package geosphere v1.5-10 (Hijmans, 2019) with distance function set to distHaversine. The correlation between the genetic and geographic distance matrices were conducted in R using the cor.test function with method set to Pearson and alternative hypothesis set to “greater” (i.e., a positive correlation). To investigate whether there was a difference in the correlation between the distances within serotype, we also performed the correlation test for a subset of the data that includes only distances among isolates form the same serotype. Pearson’s correlation test was used rather than the Mantel test as the matrices for the subsetted dataset were incomplete but a correlation among vectors was possible.

We used the package Gubbins (Genealogies Unbiased By recomBinations In Nucleotide Sequences) (Croucher et al., 2014) to investigate whether there was evidence for recombination among the isolates. The input was the SNP matrix extracted from the cgMLST scheme and default setting were used except tree inference was set to fasttree v2.1.11 (Price et al., 2010). Phandango (Hadfield et al., 2018) was used to visualize a midpoint rooted tree from Gubbins where we focus on the evidence for recent recombination.

### Virulence and Food Safety

To evaluate the pathogenicity of each isolate and whether they harbored antimicrobial resistance genes of public health interest, we used the results available at https://www.ncbi.nlm.nih.gov/pathogens/microbigge/# that uses the AMRFinderPlus tool (Feldgarden et al., 2019) to predict features from the WGS data. The prediction of phenotype from genomic data for both antimicrobial resistance and virulence genes is well-documented (Lindsey et al., 2016; Do Nascimento et al., 2017; Feldgarden et al., 2019) and as a result of the strong correlation, *in silico* prediction from genomic data is becoming standard within public health and research laboratories (Hendriksen et al., 2019).

All isolates were deposited into the NCBI Pathogen Database (see Table 1 for NCBI SRA accession), which is a large collection of isolates from various pathogens and each taxon is clustered into genetically similar sub-clusters of closely related isolates. This database currently includes 98,641 *E. coli/Shigella* isolates. Using the NCBI Pathogen Detection browser^2^ we determined whether our isolates clustered with and were genetically similar to any other environmental, food, or clinical isolates in the database.

## Results and Discussion

### Rat Sampling

We collected 254 rats from 52 alleys between March 1–June 20 and November 5–December 5, 2018. Of these, 202 rats were in adequate condition to be necropsied and tested for *E. coli* using fecal samples. All rats were presumed to be *R. norvegicus* based on morphometric measurements (Murray et al., 2020). The prevalence of *E. coli* in the sampled rats was 22% (44/202) and we were able to sequence 41 *E. coli* isolates. No *Salmonella sp.* or *Campylobacter sp.* were detected, and one *S. aureus* isolate was collected that is not discussed here.

### Pathogenicity and Antimicrobial Resistant Genes

The 41 *E. coli* isolates collected from rats throughout Chicago represented a wide genetic diversity where, based on prediction of serotype from the WGS data, there were 25 unique serotypes detected (Table 1). Seventeen serotypes had just one isolate assigned to them and four isolates could not be typed. None of the serotypes detected were of particular concern or considered threats to public health (e.g., no O157:H7 isolates or isolates containing the *eae* or *stx* genes) (Table 1). Moreover, none of the isolates appear to belong to any of the diarrheal disease causing pathotypes (STEC, EPEC, ETEC, EIEC, or EAEC) as judged by the absence of characteristic virulence markers (e.g., *stx*, *eae*, *aggR*, *eltAB*) and *ehxA*. An exception is strain CFSAN085900 that had a partial match (54% coverage, Supplementary Table 1) to the invasion plasmid antigen gene *ipaH4* that is an enteroinvasive *E. coli* (EIEC) virulence gene. Outbreaks of EIEC are rare but appear to be increasing (Herzig et al., 2019; Lagerqvist et al., 2020) yet the partial match we found requires additional research to confirm that pathotype and its prevalence within rats in the area studied. Similar results for STEC, EIEC, ETEC, and EAEC were observed in commensal Norway rats sampled in New York City (Firth et al., 2014). However, from the 133 rat samples collected from five different sites, the diarrheagenic *E*. *coli* pathotype, EPEC, was detected by PCR in 38% of fecal pellets tested (Firth et al., 2014). Le Huy et al. (2020) also found no ETEC, EPEC, or EIEC among 59 *E. coli* isolates from urban rats in Hanoi, Vietnam.

The lack of STEC isolates is not surprising given their low abundance in other studies of *E. coli* found within urban rats. Of the few studies that have tested wild rats for STEC (Espinosa et al., 2018), only 1.6% [e.g., 10 of 633 (1.6%) *E. coli* were STEC from a study of rats in Vancouver, British Columbia, Canada; Himsworth et al., 2015]. However, other studies that focused on rats near cattle farms found a higher prevalence of STEC where 20% (2/10) of rats in Denmark shed verocytotoxin-producing *E. coli* (Nielsen et al., 2004) and 40% (4/10) of rats in the Czechia shed STEC O157 (Cizek et al., 1999). These differences are likely due to proximity to cattle reservoirs. However, very few studies have assessed the prevalence of STEC in urban rats and there may be significant variation in rat pathogens between cities. More research is needed to identify the ecological factors promoting pathogen prevalence in different urban habitats.

Nearly all strains contained three AMR genes (Supplementary Table 1) that are common in *E. coli*: the multidrug efflux RND transporter permease subunit *acrF*, the nearly ubiquitous *blaEC* that provides multi-resistance to β-lactam antibiotics, and the multidrug efflux MFS transporter *mdtM* (Feldgarden et al., 2019). These AMR genes are also common among the isolates in the NCBI Pathogen Detection database and are few compared to the number of AMR genes found among *E. coli* isolated from humans or food animals (Tadesse et al., 2012) or found in isolates from food (Jans et al., 2018). One AMR gene of note is blaCMY-2 found in the lone O153:H30 isolate (Supplementary Table 1) as that gene is in the large database rare (i.e., approximately 2% of the 130,000 *E. coli* isolates have it). Therefore, the majority of *E. coli* within the rats studied here are not unique with respect to AMR resistance and, therefore, likely do not represent a vector for novel AMR genes.

When comparing the 41 isolates to the large public Pathogen Database at NCBI, we only found one isolate to cluster with isolates other than those collected as part of this study. Those other isolates were *E. coli* collected by the United States Department of Agriculture Food Safety Inspection Services (FSIS) from a swine facility that, interestingly, was also located in Illinois. The clustering of only one isolate from the rats sampled throughout the Chicago area with other isolates that are predominantly of clinical or food or food facility origin further suggests that the rats within the Chicago area may not represent a public health hazard regarding pathogenic *E. coli*, as the strains are not circulating among sickened individuals. Our sampling of rats is not exhaustive but does provide an estimate of the likelihood that pathogenic STEC *E. coli* are found in rats within the study area.

### Phylogenetics, Population Genetics, and Recombination

**FIGURE 2:**
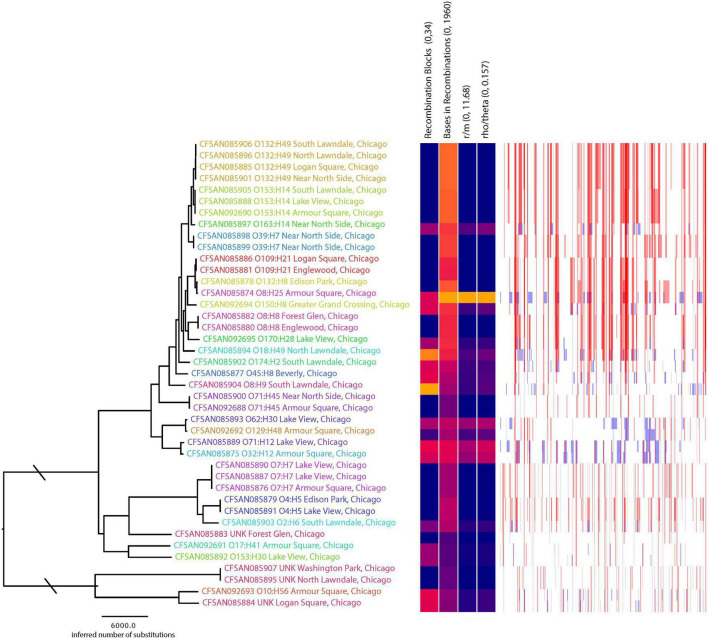
Phylogenetic tree inferred using FastTree with the matrix with recombination filtered based on the analysis with Gubbins. Column labels on the heatmap include the range of values where blue represent 0 and red represents the maximum value. Plot next to the heatmap shows the locations among the phylogeny where there is evidence of recombination; red lines indicate recombination was evidence among terminal branches where blue indicates recombination occurring among internal branches of the phylogeny. Phylogenetic tree tip labels are colored according to serotype; labels also include the serotype.

**FIGURE 3:**
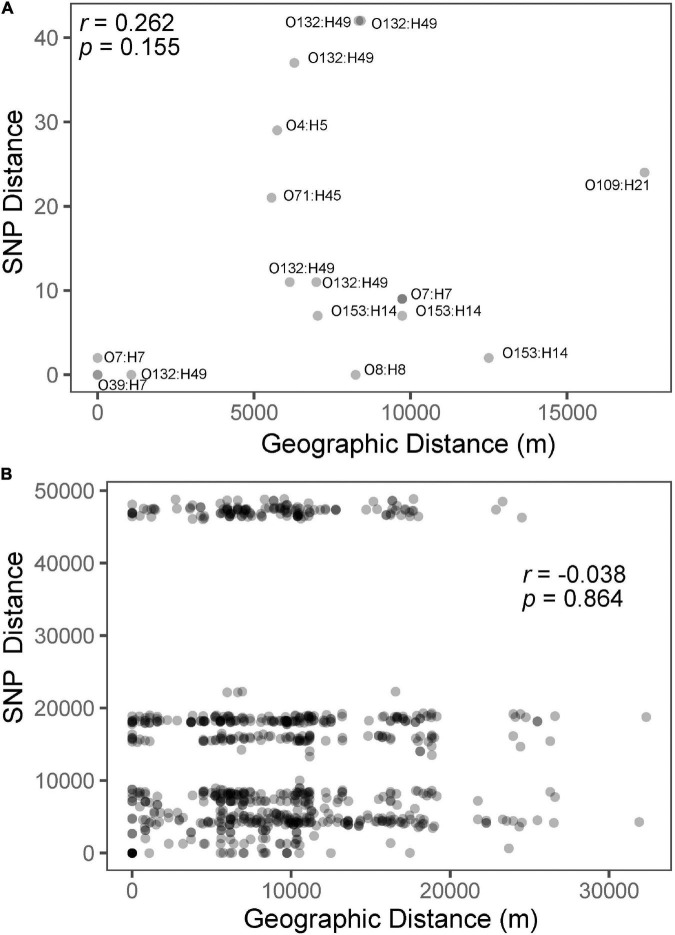
Isolation by distance plots and correlation coefficients for only those from the same serotype with points labeled according to serotype **(A)** and for all isolates **(B)**. The three horizontal groups of points are the result of pairwise comparisons between isolates from each of the three major phylogenetic clades (Figure 2).

A total of 85,529 variant positions were detected within concatenated cgMLST loci, representing approximately 1.7% of the average assembly size of 4.9 Mbp among the isolates we analyzed. This large number of variants is not unexpected given the genetic diversity observed among the isolates (e.g., 25 serotypes predicted). Phylogenetic analysis of the SNP matrix that was generated from cgMLST loci showed isolates which belong to the same serotype cluster together, regardless of whether they came from the same location. Furthermore, serotypes with a single representative isolate did not cluster according to geographic location (Figure 2). Given that isolates from the same location did not cluster together on the phylogenetic tree, it is not surprising that there was little correlation (*r* = −0.038*; p* = 0.864) between genetic differentiation and geographic distance (Figure 3B). However, when subsetting the data to only include comparisons among isolates belonging to the same serotype there was a stronger correlation (*r* = 0.262, *p* = 0.155) between the two distance measures (Figure 3A). This may suggest that what recombination and migration is occurring is at the serotype level rather than the wider species level, which in turn would support the hypothesis that *E. coli* are predominantly non-recombining distinct lineages within which there may be recombination (Tibayrenc and Ayala, 2017). A larger sample size within each serovar would help to determine the robustness of this interpretation. We also found that isolates from the same serotype for which sequence type based on MLST could be determined did not differ illustrating the similarity among isolates within serotypes (Table 1). These population genetic analyses contradict to some degree the genetic structure that would be expected among the rats from which they were sampled (e.g., stricter isolation by distance where rats from the same location would be more genetically similar to one another than they would be to rats from different populations) (Byers et al., 2019). This difference in population structure may indicate exposure to diverse sources of *E. coli* such as sewage, food waste, and pet waste.

There were 8,283 or 9% of the positions within the SNP matrix that were found to have a genealogical history indicative of recombination. Those events tended to be present among the tips of the phylogeny rather than occurring historically during primary diversification of the isolates (Figure 2). This is consistent with the picture of isolation by distance being observed within serotypes rather than among them and that serotypes represent clonal lineages. The topologies between the phylogeny inferred with the SNP matrix and that from Gubbins where sites indicative of recombination was filtered were only slightly different (Robinson-Foulds distance = 12, path difference = 54).

## Conclusion

Our investigation into the pathogenicity and population genetics of *E. coli* found within rats throughout the Chicago Metropolitan area suggests that it is unlikely those rats serve as a reservoir for STEC and thus serve as a vector for contamination of the food supply with that serotype. The diversity found was surprising where 25 different serotypes were detected. The genetic diversity among those isolates followed an isolation by distance model, however, when only considering isolates from the same serotype; recombination was evident more frequently among tips rather among internal (i.e., ancestral) branches within the phylogeny. Those two pieces of evidence support the hypothesis that serotypes of *E. coli* are predominantly clonal within which recombination may occur [i.e., nearly clonal population (NCP) and near-clading model described in Tibayrenc and Ayala (2017)]. This departure from clonality is also consistent with the emerging consensus that recombination is a major contributor to nucleotide diversity within *E. coli* (Bobay et al., 2015). Last, assuming rats follow a stricter isolation by distance model, these results suggest that rat population genetic structure and that of the *E. coli* may be independent of one another where rats within the same population harbor distinct serotypes despite being closely genetically related. Future research that directly compares the population structure of pathogens and rat hosts could help develop strategies to mitigate rats and their public health threats at biologically relevant scales.

## Data Availability Statement

The datasets presented in this study can be found in online repositories. The names of the repository/repositories and accession number(s) can be found in the article/Supplementary Material.

## Ethics Statement

The animal study was reviewed and approved by Lincoln Park Zoo.

## Author Contributions

JP performed the analyses. MM was responsible for the collection of data. All authors participated in the writing of the manuscript.

## Conflict of Interest

The authors declare that the research was conducted in the absence of any commercial or financial relationships that could be construed as a potential conflict of interest.

## Publisher’s Note

All claims expressed in this article are solely those of the authors and do not necessarily represent those of their affiliated organizations, or those of the publisher, the editors and the reviewers. Any product that may be evaluated in this article, or claim that may be made by its manufacturer, is not guaranteed or endorsed by the publisher.

## Footnotes

1
https://github.com/phac-nml/ecoli_serotyping

2
https://www.ncbi.nlm.nih.gov/pathogens/isolates/
